# Soman (GD) Rat Model to Mimic Civilian Exposure to Nerve Agent: Mortality, Video-EEG Based *Status Epilepticus* Severity, Sex Differences, Spontaneously Recurring Seizures, and Brain Pathology

**DOI:** 10.3389/fncel.2021.798247

**Published:** 2022-02-07

**Authors:** Meghan Gage, Nikhil S. Rao, Manikandan Samidurai, Marson Putra, Suraj S. Vasanthi, Christina Meyer, Chong Wang, Thimmasettappa Thippeswamy

**Affiliations:** ^1^Neuroscience Interdepartmental Program, Iowa State University, Ames, IA, United States; ^2^Department of Biomedical Sciences, College of Veterinary Medicine, Iowa State University, Ames, IA, United States

**Keywords:** nerve agent, *status epilepticus* severity, sex as a biological variable, telemetry, mixed-sex cohort, medical countermeasures

## Abstract

Modeling a real-world scenario of organophosphate nerve agent (OPNA) exposure is challenging. Military personnel are premedicated with pyridostigmine, which led to the development of OPNA models with pyridostigmine/oxime pretreatment to investigate novel therapeutics for acute and chronic effects. However, civilians are not premedicated with pyridostigmine/oxime. Therefore, experimental models without pyridostigmine were developed by other laboratories though often only in males. Following OPNA exposure, prolonged convulsive seizures (CS) or *status epilepticus* (SE) are concerning. The duration and severity of CS/SE determine the extent of brain injury in survivors even after treating with medical countermeasures (MCM)/antidotes such as atropine, an oxime, and an anticonvulsant such as diazepam/midazolam. In this study, using a large mixed sex cohort of adult male and female rats, without pretreatment, we demonstrate severe SE lasting for >20 min in 82% of the animals in response to soman (GD,132 μg/kg, s.c.). Atropine sulfate (2 mg/kg, i.m.) and HI-6 (125 mg/kg, i.m.) were administered immediately following soman, and midazolam (3 mg/kg, i.m.) 1 h post-exposure. Immediate MCM treatment is impractical in civilian exposure to civilians, but this approach reduces mortality in experimental models. Interestingly, female rats, irrespective of estrous stages, had an average of 44 min CS (stage ≥ 3), while males had an average of 32 min CS during SE, starting from soman exposure to midazolam treatment. However, in telemetry device implanted groups, there were no significant sex differences in SE severity; males had 40 min and females 43 min of continuous CS until midazolam was administered. No animals died prior to midazolam administration and less than 5% died in the first week after soman intoxication. In telemetered animals, there was a direct correlation between EEG changes and behavioral seizures in real-time. In the long-term, convulsive spontaneously recurring seizures (SRS) were observed in 85% of randomly chosen animals. At 4-months post-soman, the brain histology confirmed reactive gliosis and neurodegeneration. The novel findings of this study are that, in non-telemetered animals, the SE severity following soman intoxication was significantly greater in females compared to males and that the estrous cycle did not influence the response.

## Introduction

Chemical warfare or organophosphate nerve agents (OPNA) are threats to both civilians and military personnel worldwide. The sarin attacks in Tokyo and Syria, VX attacks in Malaysia, and Novichok attacks in England demonstrate the real threat of OPNA to civilians (Morita et al., 1995; Suzuki et al., 1995; Okumura et al., 1996; Miyaki et al., 2005; Yanagisawa et al., 2006; Zarocostas, 2017). Currently, we lack effective treatment for nerve agent survivors (Dolgin, 2013; Fields, 2017; Ciottone, 2018; Stone, 2018). Until recently, preventing acute deaths due to OPNA exposure had taken a top priority. However, reports on life-long health consequences of sarin attack survivors are beginning to emerge. Sarin exposed victims, though hospitalized and treated with medical countermeasures (MCM), developed seizures along with cognitive, motor, and psychological impairments over the long-term (Morita et al., 1995; Suzuki et al., 1995; Okumura et al., 1996; Miyaki et al., 2005; Jett et al., 2020). OPNAs are cholinesterase inhibitors and potent seizurogenic agents (Jett, 2012). In animal models, acute OPNA exposure induces *status epilepticus* (SE) and other cholinergic symptoms. The current MCMs (atropine, oxime, and diazepam/midazolam) control symptoms in experimental models but do not prevent long-term neurotoxicity, such as persistent neuroinflammation and neurodegeneration (Philippens et al., 1992; McDonough and Shih, 1997; de Araujo Furtado et al., 2012; Aroniadou-Anderjaska et al., 2016; Apland et al., 2017; Niquet et al., 2017; Wu et al., 2018; Lumley et al., 2021). Thus, there have been investigations to identify novel therapeutic targets and therapeutics in addition to MCM to prevent OPNA-induced long-term neurotoxicity. To achieve this goal, an appropriate experimental model with reproducible and quantifiable outcomes that closely mimics a real-world scenario of OPNA exposure is essential.

Nerve agents have been used to target both military and civilian populations (Haines and Fox, 2014; Rosman et al., 2014; Bajgar et al., 2015). Therefore, over the years, experimental models were developed to test interventional strategies for nerve agent exposure. Since these agents are highly regulated and permitted to use by defense or authorized high-security laboratories, diisopropylfluorophosphate (DFP) has been used as a surrogate for soman/sarin in academic laboratories (Reddy and Kuruba, 2013; Bruun et al., 2019; Gage et al., 2020; Putra et al., 2020). DFP is structurally similar to soman (Gotor et al., 2011; Reddy et al., 2021). The brain pathology caused by both DFP and OPNA induced SE are also similar (de Araujo Furtado et al., 2010; Deshpande et al., 2010; Todorovic et al., 2012; Guignet et al., 2020; Putra et al., 2020; Rojas et al., 2021). Both DFP and OPNAs irreversibly inhibit acetylcholinesterase (AChE) and cause neurological, respiratory and cardiac symptoms (Lemercier et al., 1983; Kerenyi et al., 1990; Miller et al., 1993; Grunwald et al., 1994; Auta et al., 2004). Military personnel are pre-medicated with pyridostigmine (PB), a reversible AChE inhibitor to protect from OPNAs (Kerenyi et al., 1990; Miller et al., 1993; Grunwald et al., 1994). The experimental models were developed based on the premise that PB pretreatment may be required to mimic a real-world scenario of military personnel exposure to OPNA. However, PB has poor permeability and does not protect the brain from long-term OPNA toxicity, and PB itself causes adverse effects (Kerenyi et al., 1990; Grunwald et al., 1994; Philippens et al., 1996; Coelho and Birks, 2001). Moreover, it is impractical to pre-medicate several millions of civilians with PB. Therefore, a well-characterized model without PB pretreatment would mimic a real-life civilian scenario of OPNA exposure, which may be appropriate to investigate the mechanisms involved in the onset of brain pathology and for testing the long-term neuroprotective effects of investigational new drugs (de Araujo Furtado et al., 2010; Schultz et al., 2012; Putra et al., 2020). However, immediate atropine and oxime treatment is required to minimize mortality in experimental models, which is not feasible in real-world scenario of civilian exposure to nerve agents.

In this rigorous soman study, a mixed cohort of adult male and female rats were used to demonstrate the impact of sex on initial seizure severity in response to soman (132 μg/kg, 1.2 LD_50_) without PB/MCM pretreatment to better model a real-world scenario of OPNA exposure. We used wireless telemetry device to observe real-time responses of soman-induced SE in mixed-sex cohort. Further, to test whether the initial SE severity caused spontaneously recurring seizures (SRS) and brain pathology in the long-term, in a separate cohort of animals, telemetry devices were implanted 2 months after soman exposure and the animals were video-EEG monitored continuously (24/7) for 5 weeks. The novel findings from these experiments are discussed here.

## Materials and Methods

### Animals, Care, and Ethics

The adult male and female Sprague Dawley rats (7–8 weeks old) used in this study were purchased from Charles River, United States. Male and female rats were housed individually in separate cages but in the same room. Animals had access to unlimited food and water in a 12 h day and night cycle and housed in an enriched environment. All experiments were conducted as per the approved IACUC protocols. Telemetry surgery was conducted at the Principal Investigator’s laboratory, Iowa State University, Ames, IA, United States. Soman exposure was done at MRIGlobal, Kansas City, MO, United States. All animals were euthanized at the end of the study with pentobarbital sodium (100 mg/kg, i.p.) as per the American Veterinary Medical Associations Guidelines for the Euthanasia of Animals.

### Chemicals

The MRIGlobal, Kansas City, CA, United States purchased and administered soman (>95% pure) to the animals at their designated laboratory as per approved IACUC protocol. Soman was prepared in cold 0.1 M PBS just prior to administration. Atropine sulfate (99.9% pure- by LC/MS, ATS, Thermo Fisher Scientific) and HI-6 (99.9% pure- by LC/MS, Kalexsyn, Kalamazo, MI, United States) were prepared fresh in saline at 5 and 50 mg/mL, respectively. All key chemicals, except soman, were authenticated, and purity was determined by LC-MS method at the Metabolomics Laboratory, Iowa State University, Ames, IA, United States. Midazolam (MDZ, prepared as 5 mg/mL stock solution) was supplied by MRI Global, and pentobarbital sodium for euthanasia was purchased from Iowa State University Lloyd Veterinary Medical Center Hospital Pharmacy.

We used the following antibodies for immunohistochemistry (IHC) to determine gliosis and neurodegeneration: ionized calcium-binding adaptor molecule (IBA1, goat, 1:400, Abcam, Cambridge, United Kingdom ab5076, AB_2224402) for microglia and macrophages, cluster of differentiation 68 (CD68, rabbit polyclonal, 1:300, ab125212, AB_10975465) for phagocytic microglia/macrophages, glial fibrillary acidic protein (GFAP, mouse monoclonal, 1:400 for IHC, Sigma, AB5804, AB_2109645) for astrocytes, and NeuN (rabbit, polyclonal, 1:400, Millipore, MAB377, AB_2298772) for neurons. NeuN immunostaining followed by Fluoro-Jade B (FJB, Histochem) staining was used to identify degenerating neurons. Secondary species antibodies such as Alexa Fluor conjugated (1:80), biotin-conjugated (1:400), and streptavidin-conjugated (1:300) were purchased from Jackson ImmunoResearch Laboratories West Gove, United States. All primary and secondary antibodies were diluted in PBS containing 2.5% donkey serum, 0.1% tritonX-100, and 0.25% sodium azide. Streptavidin conjugated antibodies were diluted in PBS without a detergent. FJB was diluted in 0.1% acetic acid. All antibodies were serially diluted to determine optimum concentration. In addition to primary antibody omission step during tissue processing for IHC, neutralizing antibody was used to determine the specificity of an antibody.

### Telemetry Device Implantation

We used 44 rats for telemetry device implantation (27 males and 17 females). All but nine male rats were implanted with a telemetry device 3–4 weeks before exposing to soman to determine real-time electrographic changes in the brain in response to soman injection. The nine male rats received the device implantation 2 months after the soman exposure to identify SRS. We used CTA-F40 PhysioTel™ telemetry device (Data Sciences International, St. Paul, MN, United States) for video-EEG acquisition. Implantation was performed as in our previous publications (Puttachary et al., 2016; Gage et al., 2020; Putra et al., 2020; Sharma et al., 2021). Prior to surgery, the animals were administered an analgesic, buprenorphine (0.3 mg/kg, s.c.) before the induction of anesthesia with 3.0% isoflurane (flow rate at 1 L/min O_2_) and maintained at 1.0–1.5% during surgery. We used SomnoFlo anesthetic equipment from Kent Scientific (Torrington, CT, United States). Artificial tears ointment was applied to prevent dry eyes and corneal ulceration during/after surgery. After drilling holes bilaterally, the electrodes were placed on the surface of the dura mater, overlying the cortical hemispheres, and the telemetry device was tunneled into a subcutaneous pouch. The electrodes were secured to the skull with dental cement (A-M Systems, Carlsborg, WA, United States) and the incision was closed with sterile surgical clips. Vetropolycin, a triple antibiotic ointment, was applied to the surgical site. Baytril (5 mg/kg, s.c., Bayer Pharma, Pittsburgh, PA, United States) and 1 mL of normal dextrose saline were administered subcutaneously before the animals recovered. The animals were individually caged and placed on PhysioTel receivers (RPC-1) connected to the Data Exchange Matrix 2.0 (DSI) for continuous, integrated, video-EEG acquisition using the Ponemah Acquisition software. During the 3–4 weeks post-surgery prior to soman exposure, baseline EEG was recorded for 4 days to cover both day and night cycles to evaluate the impact of surgery on brain activity for each animal. The telemetry device has a sensor to record body temperature and locomotor activity in addition to EEG activity. In the nine male rats that received telemetry implant at 2-month post-soman, EEG data was acquired for 5 weeks continuously (24/7) to identify SRS.

### Vaginal Cytology and Estrous Stage Determination

**FIGURE 1:**
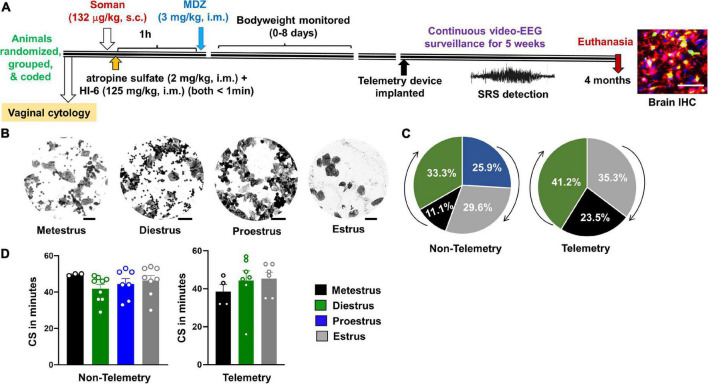
Experimental design and the relationship between estrous and convulsive seizures (CS) duration during *status epilepticus* (SE). **(A)** After randomization, grouping, and coding, three cohorts of animals were exposed to soman; first cohort had 24 rats (all males; nine rats from this group were used for implanting telemetry device at ∼3 months post-soman), second cohort had 50 rats without telemetry (25 rats per sex), and the third cohort had 35 rats with telemetry (17–18 rats per sex) and 10 rats without telemetry (eight males and two females). Each cohort of animals received soman (132 μg/kg, s.c.), HI-6 (125 mg/kg, i.m.), atropine sulfate (2 mg/kg, i.m.), and midazolam (3 mg/kg, i.m.) from the same pool of reagents prepared fresh on the day of the experiment. **(B)** Representative images showing vaginal cytology at each stage of estrous. Scale = 25 μm. **(C)** Percent of telemetry and non-telemetry animals at each stage of estrous. **(D)** Number of minutes in CS during SE for females in each stage of estrous. ANOVA or Kruskal–Wallis (*n* = 3–10).

About an hour before soman exposure, vaginal lavage and sampling were done, as described in our recent publication (Gage et al., 2020). Approximately 300 μL of sterile saline was dispensed into the vagina and flushed two—three times before aspiring it on a slide coated with chrome alum gelatin. The samples were smeared on the slide and air-dried at room temperature. The slides were stained with 0.1% cresyl violet for 1 min and washed with double distilled water twice for a minute. A light microscope (20×, Leica DMi8 with Leica K5 sCMOS camera) was used to image and determine the stage of estrous using the standard criteria described in our recent publication (Gage et al., 2020). Proestrus was characterized by nucleated epithelial cells while estrus had non-nucleated cornified epithelial cells. Metestrus and diestrus were characterized by infiltration of neutrophils; diestrus samples had a higher ratio of neutrophils to cornified epithelial cells. Representative images for each stage of the estrous cycle are presented in Figure 1B. The estrous stages of rats were unknown to the experimenters when the animals were exposed to soman.

### Soman Exposure and Medical Countermeasures Treatment

We exposed 119 rats which included 44 females and 75 male rats (44 with telemetry and the rest without telemetry, Table 1). Seven naïve controls without any treatment were used for immunohistochemistry (IHC). Out of 75 males, 51 animals were housed in the same room as the other 44 female rats. The animals were exposed to soman in three cohorts on three different days; first cohort, 24 rats (all males; 9 rats from this group were used for implanting telemetry device at 2 months post-soman); second cohort, 50 rats without telemetry (25 rats per sex), and third cohort, 35 rats with telemetry (17–18 rats per sex) and 10 rats without telemetry (8 males and 2 females). Each cohort of animals received soman (1.2 LD_50_, 132 μg/kg, s.c.), followed immediately by HI-6 (125 mg/kg, i.m.) and atropine sulfate (2 mg/kg, i.m.), and 1 h later by midazolam (3 mg/kg, i.m.) from the same pool of reagents prepared fresh on the day of the experiment. The protocol is illustrated in Figure 1. The experimental groups and sample size are tabulated in Table 1.

**TABLE 1:** Soman exposed experimental groups.

Sex	Time of telemetry device implantation	Number of animals
Males	3–4 weeks prior to soman	18
Females	3–4 weeks prior to soman	17
Males	2 months after soman	9
Males	Not implanted	48
Females	Not implanted	27

### Seizure/*Status Epilepticus* Behavioral Scoring for Severity

Animals were randomized and coded prior to soman administration. Following soman exposure, animals were scored in real-time for SE severity. Video recordings were used for secondary validation as in our previous DFP studies in the rat model (Gage et al., 2020; Putra et al., 2020). We used similar criteria as the DFP model to determine the stages of SE in soman exposed animals. The staging of seizures during SE was scored as follows: stage 1 – excessive salivation, lacrimation, urination and defecation (SLUD), mastication, chewing; stage 2 – the stage 1 signs progressed to tremors, wet-dog shakes, head nodding, neck jerks, kyphosis, and opisthotonus; stage 3 – forelimb clonus, Straub tail, rearing and rigid extension of forelimbs; stage 4 – rearing, forelimb clonus and loss of righting reflex; and stage 5 – abducted limbs clonus/repeated rearing and generalized seizures. An example of behavioral and EEG correlates of different stages of soman-induced SE and the corresponding power spectrum are illustrated in Figure 3. Stages 1 and 2 were considered non-convulsive seizures (NCS), and stage ≥ 3 were considered convulsive seizures (CS). We calculated the duration of CS (stage ≥ 3) for each animal to further classify the severity of SE as mild (<10 min) or severe (>30 min) during the 1 h from soman exposure to midazolam injection. In this study, SE severity refers to the duration of convulsive seizures (stage ≥ 3) between the first onset of CS and midazolam treatment. SE severity considers both the duration and the stage of seizure (stage ≥ 3).

**FIGURE 2:**
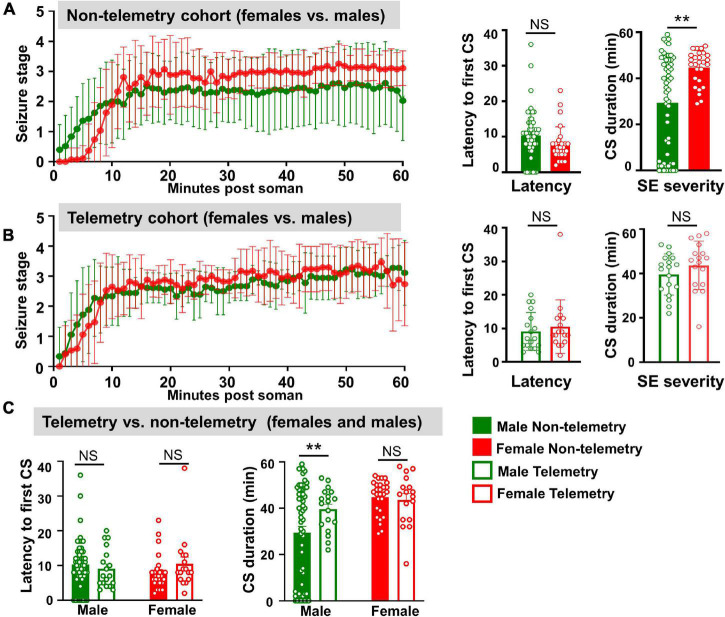
Behavioral SE severity and latency to the onset of first CS comparison between sexes and telemetry versus non-telemetry animals. **(A)** Seizure stage overtime, latency to first CS and CS duration in non-telemetry males and females. **(B)** Seizure stage overtime, latency to first CS and CS duration in telemetry males and females. **(C)** Latency to first CS and CS duration comparison between non-telemetry and telemetry animals. Mixed measures ANOVA, or Mann–Whitney (*n* = 18–57). **p < 0.01.

**FIGURE 3:**
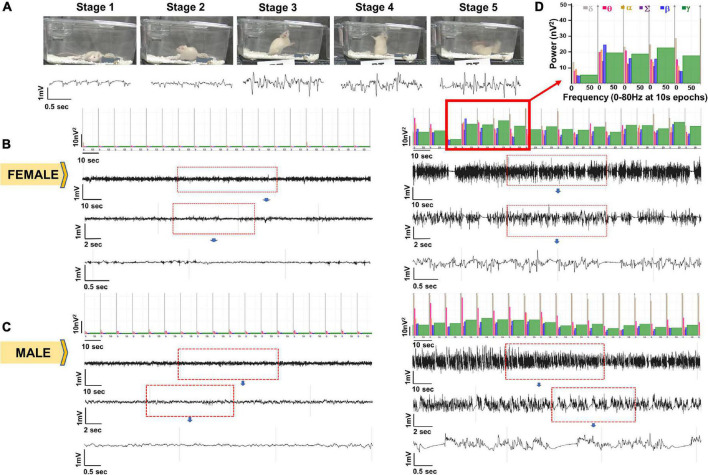
Representative EEG traces and the corresponding images captured from integrated video-EEG system. **(A)** EEG signatures for seizure stages 1–5 with corresponding power and behavior. **(B)** Baseline (left) and SE EEG traces for a female animal. **(C)** Baseline (left) and SE EEG traces for a male animal. **(D)** Enlarged power spectrum.

### Seizure/*Status Epilepticus* and Spontaneously Recurring Seizures Scoring From Integrated Video-EEG

We used the baseline EEG to normalize post-soman EEG for accurate detection of epileptiform spikes and seizures. Artifacts such as electrical noise, exploratory behavior, and grooming were identified and excluded from epileptiform spike analysis as described previously (Tse et al., 2014; Puttachary et al., 2015, 2016; Sharma et al., 2018a). All epileptiform spikes and seizures were identified using the NeuroScore 3.4.0 software. Epileptiform spikes were defined by a duration between 1 and 50 ms. The threshold for the amplitude of spikes was determined by each animals’ baseline EEG. The values were summed across groups at different time points for both male and female rats. SRS were identified using an automated seizure detection module in Neuroscore in which seizures were considered as events with spike trains lasting at least 20 s with minimum intervals of 0.05 s and maximum intervals of 1 s. NeuroScore also calculated the average duration of each seizure episode and the total time spent in a seizure with these parameters. All SRS events on EEG were manually confirmed by checking the corresponding behavioral convulsive seizure from an integrated video module and power spectrum in NeuroScore. Seizure and spike reports for each animal were generated and the data was processed for statistical analysis and graphing.

### Tissue Processing, Immunohistochemistry, and Cell Quantification

As in our previous studies, animals were perfused (60 mL/min at 80 mm Hg) with PBS followed by 4% paraformaldehyde (PFA), and the brains were isolated (Gage et al., 2020; Putra et al., 2020). Following incubation in 4% PFA for 24 h, the tissue was transferred to 25% sucrose in PBS for at least 48 h at 4°C. Brains were then gelatin embedded (15% type A porcine gelatin, 7.5% sucrose, 0.1% sodium azide) overnight at 4°C before freezing in liquid nitrogen, cooled by isopentane. Using a cryostat (Thermo Fisher), the gelatin embedded brains were sectioned (16 μm) and collected onto gelatin coated slides so that each slide contained coronal sections from rostral to caudal as described in our previous publication (Puttachary et al., 2016). Slides were stored at −20°C until they were processed for IHC.

Before the sections were processed for IHC, the brain sections were subjected to antigen retrieval by treating with citric acid solution (10 mM citric acid and 0.05% tween-20, pH 6.0) at 95°C for 23 min. After cooling, slides were placed into Shandon racks and washed with PBS for an hour and incubated in blocking buffer for an hour (10% donkey serum, 0.05% TritonX-100 in PBS) followed by incubation with primary antibodies overnight at 4°C. The next day, slides were washed for an hour in PBS and incubated with FITC conjugated or biotin-conjugated secondary antibodies for an hour. Slides were washed again for an hour with PBS before incubating with streptavidin-conjugated antibodies for an hour followed by washing with PBS for another hour. Slides were then mounted with medium containing DAPI and used for imaging. For FJB staining, following staining with NeuN and washing with PBS, slides were washed three—four times with distilled water before placing in 0.006% potassium permanganate for 5 min (Putra et al., 2020; Gage et al., 2021). Slides were again washed in distilled water three—four times before submerging in 0.0003% FJB solution for 10 min. FJB stained slides were dried and dipped in xylene for clearing before applying Surgipath acrytol. After IHC, slides were stored at 4°C.

The Leica DMi8 inverted fluorescence microscope (Wetzlar, Germany) fitted with Leica K5 passive cooled sCMOS camera system was used to image the brain sections. Representative images of the amygdala from controls and soman-exposed animals were taken to demonstrate soman-induced reactive gliosis and neurodegeneration. Experimenters were blind to the treatment groups while compiling the data. At least four sections from each animal were imaged and quantified for each staining. Only controls (not treated with soman) and animals with severe SE (>30 min) were considered in the analysis. IBA1, GFAP, and NeuN were quantified using cell profiler; the individual pipelines for each marker are described in Supplementary Table 1. CD68 (co-localized with IBA1) and FJB were quantified manually by a blind experimenter similar to our previous publications (Putra et al., 2020; Gage et al., 2021). IBA1 and GFAP positive cells were assessed for morphology. Reactive microglia (M1-like) were considered to have retracted processes and large cell bodies (average ∼11 μm diameter) and were usually positive for CD68. Non-reactive microglia (M2-like) were considered to have long processes and small cell bodies (average ∼6 μm diameter) (Streit et al., 1999). Similarly, astrocytes were also evaluated for morphology. Reactive astrocytes (A1-like) were hypertrophic and did not have long extended processes in contrast to non-reactive astrocytes (A2-like) (Li et al., 2019). Experimenters who were blind to the experimental groups analyzed the relative size of each cell’s soma and processes and estimated the degree of morphological polarization in glial cells. It should be noted, however, that glial cells exist on a spectrum of reactive to non-reactive, and this analysis is limited to a certain degree of subjectivity.

### Experimental Design, Statistics, and Rigor

The mixed cohort of male and female animals were randomized, ignoring sex, and the experimental groups were blinded until the data were completely analyzed. The normality of the data was evaluated with Shapiro–Wilk test. Data was graphed and statistics were performed by Graphpad version 9.0; specific tests are outlined in the corresponding figure legends. We had taken measures to minimize variables: (i) seizure severity during the SE was quantified by both direct observation and offline video analysis by at least two independent observers; (ii) authentication of the identity and purity of key MCMs by LC-MS; (iii) SRS events on EEG were also manually verified with integrated video module for behavior and power spectrum in NeuroScore; and, (iv) determination of the optimum concentration of the primary antibodies by serial dilution and their identify was confirmed by using neutralizing primary antibodies.

## Results

### Female Animals, Irrespective of Estrous Stages, Responded to Soman More Consistently Than Males in Both Telemetry and Non-telemetry Groups

The experimental design for soman exposure is illustrated in Figure 1A. The experimental groups and sample size are tabulated in Table 1. The studies from other laboratories with or without pretreatment (summarized in Supplementary Table 2) gave us a starting point for soman dose. Instead of testing different routes and doses of soman, based on previously published work from other laboratories (Schultz et al., 2012, 2014; Lumley et al., 2019), we tested 132 μg/kg subcutaneously in a batch of adult male rats and then tested in mixed sex cohorts.

About an hour before exposure to soman, vaginal cytology was conducted to determine the stage of the estrous cycle for each female rat in both telemetry and non-telemetry groups. Representative images of vaginal cytology for each stage of the estrous cycle are illustrated in Figure 1B. The percent of animals at different stages of estrous cycle at the time of soman exposure, and the total duration of CS during SE (severity) in both telemetry and non-telemetry groups are shown in Figures 1C,D. In the non-telemetry group (*n* = 27), there were 11.1% of animals in metestrus, 33.3% of animal in diestrus, 25.9% of animals in proestrus, and 29.6% of animals in estrus. In the telemetry group (*n* = 18), there were 23.5% of animals in metestrus, 41.2% of animals in diestrus, 35.3% of animals in estrus, and no animals were in proestrus at the time of exposure to soman (Figure 1C).

The telemetry group included the animals that were implanted with telemetry devices 3–4 weeks before soman exposure (to investigate the acute effects in real-time). All animals in each group were housed in the same room, male and female side-by-side but in separate cages. Each mixed-sex cohort was treated with soman (132 μg/kg, s.c.), atropine sulfate (2 mg/kg, i.m.), HI-6 (125 mg/kg), and 1 h later midazolam (3 mg/kg. i.m.) from the same reconstituted drug pools. Unlike some other soman models, the animals were not pretreated with PB or an oxime.

Surprisingly, in contrast to a general perception that female rats respond differently and inconsistently to soman/chemoconvulsants than males, in this study, female rats responded to soman more consistently, irrespective of their estrous stages, in both telemetered and non-telemetered groups (Figures 1D, 2). Seizures were scored immediately after soman until midazolam was administered (1 h duration). In non-telemetry animals, there was significant increase in CS duration in females compared to males; the average duration of CS in males was 29 min, compared to 44 min in females (Figure 2A). In the telemetry implanted group, there were no significant sex differences in SE severity; the average CS duration in males was 40 and 43 min in females (Figure 2B). Telemetry implanted males had longer durations of CS compared to non-telemetry implanted males; this was not true of females (Figure 2C). There were also no differences in latency to the onset of first CS between sexes in both telemetry and non-telemetry groups or between telemetry and non-telemetry animals (Figures 2A–C).

For the male-only cohort (1), out of 24 animals, 3 (12%) animals did not respond to Soman, 5 (20%) had <10 min CS, 2 (8%) had 10–20 min CS, and 14 (60%) had >20 min CS. In the mixed sex cohort of non-telemetry animals (cohorts 2 and 3), out of 33 males, 5 (15%) did not respond to soman, 4 (12%) had <10 min of seizures, 1 (3%) had 10–20 min, and the 23 (70%) had ≥20 min of seizures. In contrast, all non-telemetered females had ≥20 min of seizures. In telemetry animals (mixed cohort 3), all 18 males had ≥20 min, and 16 females had ≥20 min of seizures (one female had 16 min). No animals died prior to midazolam administration and <5% of animals died within 1 week of soman administration.

### Integrated Video-EEG Analysis of Soman Response in Real-Time in Male and Female Rats

Seizure severity determines the extent of brain injury and the onset of spontaneously recurring seizures (SRS) (Klitgaard et al., 2002; Nairismägi et al., 2004; Puttachary et al., 2015, 2016). However, behavioral seizure quantification alone does not reveal whether the seizures arise from the brain or due to the peripheral effects of soman. To account for this, we quantified the duration of CS *via* EEG during 1h between soman and midazolam administration. Representative EEG traces and the corresponding images captured from integrated video-EEG system, and the power spectrum are illustrated from both male and female rats (Figure 3). Behavioral seizures observed on videos correlated with changes in EEG such as high frequency and high amplitude spikes with a corresponding increase in the gamma power.

**FIGURE 4:**
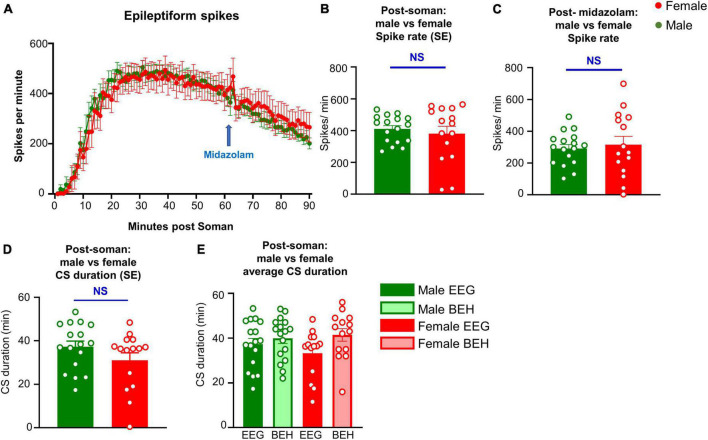
Seizures (CS, convulsive seizure) detected on EEG and spike rate comparison between male and female rats during SE, and the immediate effect of midazolam (3 mg/kg, i.m.) on spike rate. **(A)** Spike rate over time between male and female animals. **(B,C)** Average spike rate during soman **(B)** and in the first 30 min after soman. **(D)** Total duration of seizures post soman. **(E)** SE duration comparison between behavioral (BEH) SE and EEG based SE in males and females.

Seizures detected on EEG and spike rate during SE were compared between male and female rats. There were no significant differences between male and female rats in the overall CS duration and rate of epileptiform spikes during SE (Figures 4A,B,D). We also calculated the spike rate in the first 30 min post-Soman; there were no significant sex differences (Figure 4C). When compared between the behavioral seizures (CS) observed by experimenters and the seizures detected by EEG, there were no significant differences in either sex though there were a few minutes higher in behavioral SE score by experimenters (Figure 4E).

### Soman Exposure Induced Epileptogenesis: Spontaneously Recurring Seizures, Epileptiform Spikes, Gliosis, and Neurodegeneration

**FIGURE 5:**
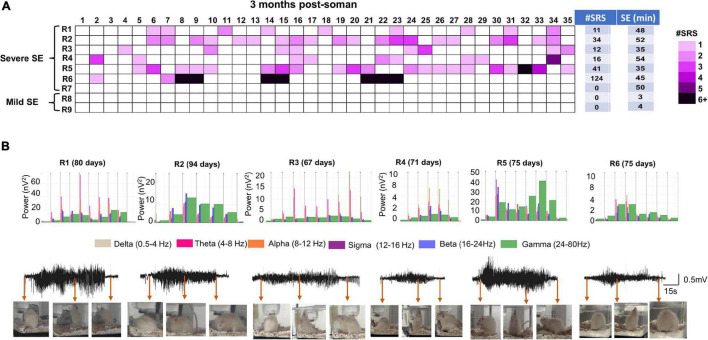
Spontaneous recurrent seizures beginning 2 months post Soman. **(A)** Heatmap showing the incidence of SRS in male rats during the 35 days of continuous video-EEG observation. Telemetry device was implanted at ∼2 months post-SE. Six out of 7 severe SE rats developed SE. Two mild-SE rats did not show any SRS. Initial SE severity and the number of SRS episodes during the observation period is tabulated next to the heatmap. **(B)** Representative SRS episode and corresponding behaviors captured from integrated video-EEG system, corresponding powerbands are illustrated. R1–R6 represent rat number and the day in parenthesis indicate the SRS occurrence post-soman.

It is important to note that there was no significant difference in SE severity between male and females in telemetered animals, unlike in non-telemetered animals (Figures 2A,B). Knowing that the initial SE severity, irrespective of sex, induces epileptogenesis in the majority of the animals, we investigated the occurrence of SRS, gliosis, and neurodegeneration in randomly chosen male rats. We implanted telemetry devices in seven severe (≥30 min) and two mild (3–4 min) SE rats at 2 months post-soman and continuously monitored video-EEG for 5 weeks. The number of SRS episodes observed during the 35 days in each rat is represented in a heatmap (Figure 5A). There were no apparent patterns of SRS occurrence among the rats. The duration of initial SE for each animal and the total numbers of SRS per animal during the 35 days are also tabulated. Six out of seven rats from the severe group had >10 convulsive SRS during the analysis period. One rat with 50 min CS during SE did not show any convulsive SRS but did show non-convulsive SRS (not quantified) and was therefore epileptic. None of the mild SE rats had convulsive SRS. Representative EEG traces of SRS episodes and their corresponding behaviors and power spectrum are shown in Figure 5B.

**FIGURE 6:**
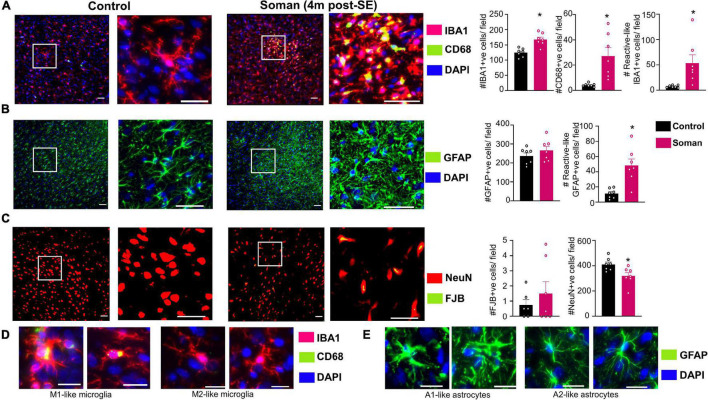
Representative images of amygdala (4 months post-soman) showing **(A)** reactive microgliosis (IBA1 + CD68, red IBA1 positive microglia, green/yellow CD68), **(B)** astrogliosis (GFAP, green), and **(C)** neurodegeneration (FJB + NeuN, green label FJB, red labeled cells NeuN). Examples of reactive and non-reactive astrocytes and microglia are shown in **(D,E)**. *T*-test (*n* = 7) scale bar, 50 μm **(A,B)**, 20 μm **(D,E)**; field = 0.44 μm^2^. *p < 0.05.

Immunohistochemical analysis in the amygdala for microglia/macrophages (IBA1), astrocytes (GFAP), and neurodegeneration (NeuN + FJB/NeuN) confirmed that the epileptic brains, compared to controls, had increased gliosis and neurodegeneration. Soman treated animals, compared to controls, had more IBA1 and CD68 positive cells as well as IBA1 positive cells with reactive morphology (Figure 6A). Examples of reactive and non-reactive microglia (IBA1 positive cells) are represented in Figure 6D. Interestingly there were no statistical difference in the number of GFAP positive cells but there were more GFAP positive cells with reactive morphology in soman treated animals compared to controls (Figure 6B). Examples of reactive and non-reactive astrocytes (GFAP positive cells) are represented in Figure 6E. Soman treated animals did not have a significant upregulation of FJB positive cells but did have a significant reduction in NeuN positive cells suggesting loss of neurons (Figure 6C).

## Discussion

Traditionally, chemical, biological, radiological, and nuclear agents were viewed as threats to only the military until the events of September 11, 2001, and the anthrax mailing incidents in October 2001. These incidents, as well as the sarin attacks in Tokyo and Syria, VX attacks in Malaysia, and Novichok attacks in England revealed the vulnerability of civilians to unconventional terrorism threats such as nerve agents (Morita et al., 1995; Suzuki et al., 1995; Okumura et al., 1996; Miyaki et al., 2005; Yanagisawa et al., 2006; Zarocostas, 2017; Jett et al., 2020; Yeung et al., 2020). Until recently, preventing acute deaths due to OPNA exposure had taken a top priority. There is no effective treatment available for nerve agent survivors (Dolgin, 2013; Fields, 2017; Ciottone, 2018; Stone, 2018). Consequently, strategies to develop MCMs to support civilian health preparedness and disaster response to nerve agent exposure took priority. Further, developing an appropriate animal model that closely mimics the real-world scenario for testing novel MCMs and disease-modifying agents for civilian-focused biodefense gained importance in recent years. Therefore, the present study, and the studies from other laboratories, in the rat soman model is timely and appropriate.

Several experimental models have been developed, tested, and refined over the years to more closely approximate real-world scenarios of OPNA exposure to military and public (Schultz et al., 2014). For example, some studies expose animals to agents like soman and sarin as well as DFP, which is less potent but is often used as a surrogate for real nerve agents (McDonough et al., 1998; de Araujo Furtado et al., 2010; Bruun et al., 2019; Lumley et al., 2019; Putra et al., 2020; Gage et al., 2021). Organophosphates bind to the esteratic site of AChE and inactivate the enzyme leading to both muscarinic and nicotinic receptor depolarization (McDonough and Shih, 1997; Čolović et al., 2013; Sirin and Zhang, 2014). OP antidotes or prophylaxis such as pyridostigmine bromide (PB) have been developed based on its reversible inhibition of AChE (Fulco et al., 2000). When used as a pretreatment, AChE bound to PB would be protected from irreversible inhibition by OPNAs. Therefore, the experimental models of DFP, and its surrogate soman protocols, include PB as a pretreatment strategy, and in some models, oximes (HI-6 and 2-PAM) are used (McDonough et al., 1998; Shih et al., 1999; de Araujo Furtado et al., 2010). Oximes reactivate AChE before the enzyme ages (Kerenyi et al., 1990; Dadparvar et al., 2011). The rationale for pretreatment with PB or oximes in animal models has been based on the premise that military personnel are premedicated with PB as prophylaxis. In 2003, the United States Food and Drug Administration (FDA) approved PB as a pretreatment in humans against the lethal effects of soman (Haigh et al., 2010). Interestingly, recent experimental evidence suggests that PB pretreatment has no effect on DFP-induced seizures or 24-h survival in the rat model (Bruun et al., 2019), suggesting its limited effect on the brain. In contrast, PB pretreatment protected AChE activity in the marmoset diaphragm despite exposure to a dose of soman that would be lethal in unprotected animals (Haigh et al., 2010). PB does not effectively cross the BBB, and its side effects are well known (Kerenyi et al., 1990; Shih et al., 1999). These studies from a rat DFP model and marmoset suggest PB is only effective in restoring AChE levels in peripheral organs and can be protective, but not in the brain. For long-term neurotoxicity studies, a different approach is required to minimize mortality and to mimic a real-world scenario of OPNA exposure in civilian populations. Several studies from other laboratories have developed animal models to address this issue (Schultz et al., 2012; Aroniadou-Anderjaska et al., 2016; Chaubey et al., 2017). In this rat soman study without PB pretreatment in a mixed sex cohort, we demonstrated no morality during SE despite severe convulsive seizures. The initial SE severity [i.e., the duration of convulsive seizures (stage ≥ 3) between OP exposure and midazolam treatment] determines epileptogenesis and brain pathology (Wu et al., 2018; Gage et al., 2021; Rojas et al., 2021). This is an important factor for testing disease-modifying agents for their long-term effects on the brain in animal models.

Apart from pretreatment, the lethal dose (LD_50_) of soman determines SE severity and mortality if no MCMs were administered. Several soman studies have tried to balance both mortality and seizure severity even at higher LD_50_ by utilizing a pretreatment approach. A summary of different soman models and the source is tabulated in Supplementary Table 2. In some studies, animals were treated with HI-6 before exposure to soman (McDonough et al., 1998; Shih et al., 1999). Despite pretreatment with HI-6, SE severity, or brain pathology induced by soman was unaffected implying HI-6 had no effects on brain but may have protected peripheral organs. There is no perfect model to mimic the real-world scenario for civilian population. In our model, without pretreatment, atropine sulfate and oxime HI-6 dimethanesulfonate (HI-6 DMS) were administered immediately (<1 min) after soman injection, which is impractical in a real-world scenario of OPNA exposure. The rationale for this approach was to reduce mortality by protecting the vital organs such as lungs and heart, achieved by anticholinergic effects of atropine. In this study, we used oxime HI-6 DMS instead of 2-pyridine aldoxime methyl chloride or pralidoxime (2-PAM). 2-PAM is the only FDA-approved oxime while HI-6 is not. Interestingly, unlike HI-6, 2-PAM is not effective for nerve agents such as soman (Puu et al., 1986; Sidell et al., 1997; Worek et al., 2004). However, both oximes are expected to mitigate both muscarinic receptor-mediated symptoms (lacrimation, salivation, diarrhea, miosis, and bradycardia) and nicotinic receptors mediated symptoms (tachycardia, hypertension, convulsions/tremors, and skeletal muscle paralysis) of OPNA exposure (Abdollahi and Karami-Mohajeri, 2012). HI-6 has a better brain PK kinetics than 2-PAM and targets both muscarinic (centrally) and nicotinic receptors (peripherally) effectively, and reduces mortality by two-fold. 2-PAM lacks anti-nicotinic effects (Lorke et al., 2009; Soukup et al., 2011). HI-6 has a potent spasmolytic effect due to rapid reactivation of gut AChE (Marquart et al., 2019). This would facilitate better absorption of orally active drugs, used as a follow-on therapy to achieve long-term protection, as well as nutrients to promote rapid weight gain. Despite the use of these MCMs in this study, animals had severe SE in response to soman, and no mortality was observed prior to MDZ administration, which is remarkable. Notably, no experimental model mimics real-world scenarios or human disease. However, this model is closer to the civilian exposure, than the military, but is not a perfect model as animals were treated immediately with atropine and oxime to reduce mortality.

Benzodiazepines such as diazepam and midazolam control SE and reduce mortality but do not protect SE-induced neuropathology unless given in <30 min of SE onset (Goodkin et al., 2008; Pibiri et al., 2008; Qashu et al., 2010; Todorovic et al., 2012; Iyer et al., 2015), which may not be possible to achieve in real-life scenarios of mass OPNA intoxication. Both diazepam and midazolam also reduce mortality, but they do not control epileptiform discharges and non-convulsive seizures (NCS) in OPNA and DFP models of neurotoxicity (Todorovic et al., 2012; Reddy and Kuruba, 2013; Apland et al., 2014; Lumley et al., 2019; Putra et al., 2020; Supasai et al., 2020). In this study, at 1h post-MDZ (3 mg/kg MDZ), >95% animals survived which is similar to the findings by Lumley et al. (2019). In the latter study, they also administered 3 mg/kg MDZ but at 40 min post-soman. The other three soman studies without pretreatment tested the efficacy of diazepam (10 mg/kg) with 0.8–1.0 LD_50_ soman (instead of 1.2 LD_50_), however, the mortality details were not reported (Schultz et al., 2012, 2014; Getnet et al., 2018). Recently, we also demonstrated in the rat DFP model that midazolam is more effective than diazepam in preventing mortality (Gage et al., 2021). In our soman model, midazolam was given 1-h post-exposure. In animals that had severe SE, 6/7 had several convulsive SRS and 7/7 had non-convulsive SRS during the 5 weeks of continuous video-EEG monitoring. Previous soman studies in adult Sprague-Dawley rats have also reported SRS in soman model (Schultz et al., 2014; Lumley et al., 2019). The two mild SE animals did not have convulsive SRS. In contrast, 67% of animals that received 3 mg/kg MDZ were reported to have had SRS in Lumley et al. (2019) study. Furthermore, in the latter study, the initial SE severity and duration were not correlated with SRS onset. Several studies have reported soman-induced brain pathology (McDonough et al., 1998; Apland et al., 2010, 2017; Schultz et al., 2014). In this study, all the rats that had severe SE and SRS onset showed significant reactive gliosis and neurodegeneration, the most common features of epileptogenesis. Seizures compromise blood-brain-barrier integrity and causes infiltration of circulating monocytes/macrophages into the brain (Zattoni et al., 2011; Varvel et al., 2016; Han et al., 2019). Macrophages also express IBA1 and contain CD68 (Ramprasad et al., 1996). Therefore, in this study, IBA1 + CD68 positive cells may also include macrophages.

Sex as a biological variable has been explicitly addressed in this study. We used a mixed-sex cohort of rats that were housed individually next to each other in the same room to mirror the real-world scenario of male and female populations. Vaginal cytology, an hour before soman exposure, suggested the animals were at different stages of estrous cycle. Notably, irrespective of the stages of the estrous cycle, almost all female rats used in this study had severe SE (an average of 44 min) in response to the same dose of soman used in age-matched male rats (132 μg/kg). This contradicts our previous study using DFP, which showed an increased resistivity in females compared to males (Gage et al., 2020). In our previous DFP study, males and females were tested separately and with different batches of DFP (freshly prepared) which may have had impacted the seizure response. In our current soman study, both males and females were tested as mixed cohorts at the same time with the same pool of freshly prepared soman. Another study using sarin found some sex differences in the plasma levels of cholinesterase but did not find a significant difference in LD_50_ between males or females in any stage of estrous (Smith et al., 2015). These studies suggest possible model differences between OPNAs with respect to the impact of sex. In a study on soman poisoning in rats treated with physostigmine 30 min before soman exposure, females had 50% less LD_50_ (47 μg/kg) than weight-matched male rats (92 μg/kg) (Sket, 1993). The behavioral signs of soman poisoning measured in the Sket (1993) study were chewing and body tremors which we considered as non-convulsive stages 1 and 2 seizures on a modified Racine scale in our study. In our model, we did observe higher CS duration in females compared to age-matched male rats in response to 1.2 × LD_50_ (132 μg/kg) soman, irrespective of the stages of estrous cycle. However, in telemetry implanted female and male rats, there were no significant differences in SE severity. In the rat and mouse kainate models of epilepsy, we had previously shown that surgery reduces the threshold for SE onset in males (Sharma et al., 2018b; Tse et al., 2021). In this soman study too, we observed a similar finding; telemetry male rats showed increased CS duration during SE (40 min) compared to non-telemetry male rats (32 min) (Figure 2C). In contrast, there were no differences in female rats between telemetry versus non-telemetry, possibly because non-telemetry animals had reached the maximum seizure threshold that an animal could spend in SE prior to MDZ with no mortality. This could also suggest that females are resistant to surgery induced stress though this requires further investigation.

In the Sket (1993) study, the dose of HI-6 (25 mg/kg) used in combination with atropine sulfate (80 mg/kg) was five times less than our study (125 mg/kg). However, surprisingly, they used a dose of atropine sulfate 40 times higher than normally used (2 mg/kg) in most OPNA models (Supplementary Table 2) but they could only achieve 60% protection. In our studies, HI-6, atropine sulfate resulted in no mortality during SE. In another rat study, HI-6 was more effective in females than males (Lundy et al., 1989), which may explain why there was no mortality in females in the present study during SE or immediately after midazolam treatment. HI-6 and atropine preserve more AChE activity in the skeletal muscle but had no effect on brain AChE (Sket, 1993), suggesting the need for the development of MCMs that cross the BBB to target the brain. There were no other mixed-sex cohort rat studies in soman model without pretreatment at the time of our current publication. The only other study was in the plasma carboxylesterase knockout (Es1–/–) mice, which like humans, lack plasma carboxylesterase (Duysen et al., 2011). Interestingly, female Es1–/– mice in estrous required greater LD_50_ of soman compared to the females in proestrus or with males (Kundrick et al., 2020). In our study, we did not find any estrous stage-specific differences in their response to soman exposure. Interestingly, none of the telemetry implanted female rats were in proestrous when the soman was administered.

## Conclusion

The findings from this study demonstrate that in an age-matched, mixed sex cohort, SE severity in females was greater than in males in response to soman exposure without pyridostigmine or oxime pretreatment. Telemetry device implanted males showed increased response to soman compared to non-telemetry animals. In females, there were no significant difference in SE severity (seizure stage ≥ 3 and duration) in response to soman when compared between telemetered and non-telemetered animals. There were also no sex differences in SE severity in telemetered male and female animals. The stage of the estrous cycle had no impact on SE severity in both telemetry and non-telemetry groups. Long-term continuous video-EEG recordings and brain histology confirmed that soman-induced severe SE without pretreatment can promotes epileptogenesis as evidenced by SRS occurrence, reactive gliosis, and neurodegeneration. This study demonstrates, similar to previous work by others, that soman exposure without pretreatment can lead to severe SE and the development of SRS and neuropathology. The other advantage of the model in this study is the low mortality (<5%). Considering these advantages, this model can be used in the future studies to evaluate the efficacy of potential disease modifying agents.

## Data Availability Statement

The raw data supporting the conclusions of this article will be made available by the authors, without undue reservation, to any qualified researcher.

## Ethics Statement

The animal study was reviewed and approved by the Iowa State University and the MRI Global Animal care and use committees.

## Author Contributions

TT conceptualized the idea, secured funding for the project, designed the study, and wrote the manuscript. All co-authors conducted the experiments, acquired and analyzed the data, and edited the manuscript. MG, MP, NR, SV, and CM conducted surgery to implant telemetry devices and quantified behavioral SE. SV contributed Figures 1B–D and Supplementary Table 2. MS contributed Figure 2. NR contributed Figures 3, 4. MG conducted long-term experiments and contributed Figures 5, 6. CW offered statistical support. All authors edited and approved the manuscript.

## Conflict of Interest

The authors declare that the research was conducted in the absence of any commercial or financial relationships that could be construed as a potential conflict of interest.

## Publisher’s Note

All claims expressed in this article are solely those of the authors and do not necessarily represent those of their affiliated organizations, or those of the publisher, the editors and the reviewers. Any product that may be evaluated in this article, or claim that may be made by its manufacturer, is not guaranteed or endorsed by the publisher.
