# Supplementary material for: Soman (GD) Rat Model to Mimic Civilian Exposure to Nerve Agent: Mortality, Video-EEG Based Status Epilepticus Severity, Sex Differences, Spontaneously Recurring Seizures, and Brain Pathology
Source: Front Cell Neurosci. 2022 Feb 7;15:798247. doi: 10.3389/fncel.2021.798247 (PMC8859837; doi:10.3389/fncel.2021.798247)
Supplement: Supplementary file 1 [file Table_1.DOCX]

**Table S1.** Cell profiler pipeline specifications for immunopositive cell quantification (field counted= 0.44μm^2^).

| **Marker** | **Diameter min (pixels)** | | **Diameter max (pixels)** | **Thresholding strategy** | **Thresholding**  **method** |
| --- | --- | --- | --- | --- | --- |
| GFAP | | 26 | 100 | Global | Otsu |
| IBA1 | | 28 | 150 | Adaptive | Robust background |
| NeuN | | 17 | 120 | Adaptive | Otsu |
