# Supplementary material for: Soman (GD) Rat Model to Mimic Civilian Exposure to Nerve Agent: Mortality, Video-EEG Based Status Epilepticus Severity, Sex Differences, Spontaneously Recurring Seizures, and Brain Pathology
Source: Front Cell Neurosci. 2022 Feb 7;15:798247. doi: 10.3389/fncel.2021.798247 (PMC8859837; doi:10.3389/fncel.2021.798247)
Supplement: Supplementary file 2 [file Table_2.docx]

**Table S2. Comparison of different models of soman.**

| **Model #** | **Strain/Sex/age** | **Pretreatment** | **Soman (GD)** | **Atropine** | **Oxime** | **SE severity** | **mortality** | **MDZ/DZP** | **References** |
| --- | --- | --- | --- | --- | --- | --- | --- | --- | --- |
| 1 | Male  Albino Wistar rats | Not known | 90 μg/kg  i.m. or 80% LD_50_ | Atropine sulfate  21 mg/  kg i.m. | HI-6  39 mg/kg  i.m. | No data | 1 within 24 hrs | No data | Kassa *et al.,* 2011 |
| 2 | Male Cri:CD(SD)BR rats | Pyridostigmine  131 µg/kg i.m. | 47 µg/kg i.v. | Atropine sulfate  16 mg/kg i.m. | HI-6 or 2-PAM 100 µmol/kg i.m. | No data | No data | No data | Anderson *et al.,* 1992 |
| 3 | Male New Zealand white rabbits | No data | 26.8 µg/kg or 2 LD_50,_ i.m. | Atropine sulfate  5 or 13 mg/kg i.m. | PAM, HI-6 50 µmol/kg i.m. | No data | Seen | No data | Koplovitz & Stewart 1992 |
| 4 | Male Sprague-Dawley rats | HI-6 125 mg/kg i.p. | 225 µg/kg s.c | Atropine methylnitrate 2 mg/kg i.m. | Given before soman | Soman-intoxicated animals seized for the 7 hours  (420 minutes) | 100% in > 24hrs | No data | Winkler *et al.,* 2017 |
| 5 | Male Sprague-Dawley rats | HI-6 125 mg/kg i.p. | 110 µg /kg s.c | Atropine sulfate  2 mg/kg i.m. | Given before soman | 28.9% (n = 11) of rats that developed SE (n = 38) experienced electrographic SRS | 13 of 49 animals died | DZP 10 mg/kg s.c., 30 post- SE | De Araujo Furtado *et al.,* 2010 |
| 6 | Male Crl:CDBR Vaf/Plus Sprague-Dawley rats | HI-6 125 mg/kg i.p. | 180 µg /kg s.c or 1.6 LD_50_ | 5-40 min post-SE AED or atropine i.p | Given before soman | Convulsions noticed | 40-90% | AEDs | Shih *et al.,* 1999 |
| 7 | Male Sprague-Dawley rats | HI-6 125 mg/kg i.p. | 180 µg /kg s.c or 1.6 LD_50_ | Atropine methylnitrate  2 mg/kg i.m. | Given before soman | One of the 12 animals did not show seizures on EEG but showed signs of soman poisoning | 9/12  survived to the 24-h time point | No data | Thomas and Shih 2014 |
| 8 | Male C57Bl6/J mice, 12-13 weeks | HI-6 50 mg/kg i.p. | 172 µg /kg s.c. | Atropine methylnitrate  2 mg/kg i.p. | Given before soman | SRS events were observed in 100% of surviving mice | 9 out of 20 | DZP 5 mg/kg s.c., 2h post-SE | McCarren *et al.,* 2020 |
| 9 | Adult male Sprague-Dawley rats, 3 months | HI-6 125 mg/kg i.p. | 154 µg /kg s.c.or 1.4 LD_50_ | Atropine methylnitrate (AMN) 2 mg/kg i.m. | Given before soman | Spike activity  reported | No data | MDZ 2 mg/kg  i.m. | Reddy *et al.,* 2020 & 2021 |
| 10 | Male adult Sprague-Dawley rats | No pretreatment | 0.8 or 1.0 LD_50_ | Atropine sulphate 2 mg/kg I.m. | HI-6 93.6mg/ kg i.m. | 42% of the animals had seizure | No data | DZP 10 mg/kg i.p | Getnet *et al*., 2018 |
| 11 | Male adult Sprague-Dawley rats  Telemeterized | No pretreatment | 132 µg/kg s.c. or 1.2 LD_50_ | Atropine sulphate 2 mg/kg i.m. | HI-6 93.6 mg/ kg i.m. | Seizure stages reported | No data | DZP 10 mg/kg i.p  30 min post | Schultz *et al*., 2012 |
| 12 | Male adult Sprague-Dawley rats  Telemeterized | No pretreatment | 132 µg/kg s.c. or 1.2 LD_50_ | Atropine sulphate 2 mg/kg i.m | HI-6 93.6 mg/kg i.m. | Seizure stages reported | No data | DZP 10 mg/kg i.p  30 or 40 min post | Schultz et al., 2014 |
| 13 | Male Sprague-Dawley rats, 7-8 weeks | HI-6 125 mg/kg i.p. | 154 µg/kg s.c.or 1.4 LD_50_ | Atropine sulphate 2 mg/kg i.m. | given before soman | SE duration reported (609.4 ± 37.3 minutes, n = 4) | 2 out of 23 | DZP 10 mg/kg i.m. | Apland *et al*., 2014 |
| 14 | Male Sprague-Dawley rats | No pretreatment | 132 µg/kg s.c. or 1.2 LD_50_ | Atropine sulphate 2 mg/kg i.m. | HI-6 118.5 mg/kg i.m. | 25 SRS with GD + MDZ 3 mg/kg | Mortality observed  50-70% with 1mg/kg; 10-15% with 3 or 9mg/kg MDZ | MDZ(1, 3, or 9 mg/kg) i.p. or Ketamine (10, 30, 60 or  90 mg/kg) i.p.or Valproic acid (90 mg/kg) i.p. | Lumley *et al*., 2019 |
| 15 | Male and female Es1^−/−^ mice, 8-9 weeks | No pretreatment | 82 μg/kg s.c. | Atropine sulphate 4 mg/kg i.p. | HI-6 50 mg/kg i.p. | Es1^−/−^ mice that received 1 mg/kg  MDZ after GD exposure did not survive | 1, 3, and 9mg/kg MDZ showed 18.2%,  46.2%, and 78.6% survival rates | MDZ 1,  3, or 9 mg/kg i.p. | Kundrick *et al*., 2020 |
| 16 | Male Sprague Dawley rats | HI-6 125 mg/kg i.p. | 180 µg /kg s.c | Not reported | Given before soman | Continuous epileptiform seizures that lasted in excess of 4 hr | Not reported | Not reported | McDonough *et al.,* 1998 |
| 17 | Male Sprague-Dawley rat | No pretreatment | 74.4 µg /kg s.c or 1.2 LD_50_ | Atropine sulphate 0.5 mg/kg i.m. | HI-6 125 mg/kg i.p. | 80% developed SE (16/20). | 71.5%  (5/7) survived | MDZ 3 mg/kg  i.m. | De Araujo Furtado *et al.,* 2020 |
| 18 | Male and female Sprague-Dawley rat | 17 mg/kg atropine sulphate i.p. 30 min prior to GD; HI-6 125 mg/kg i.p. 1 min prior to GD | 110 µg /kg s.c | Given before soman | Given before soman | Not reported | Seen | Not reported | Lundy *et al*., 1989 |
| 19 | Male Sprague Dawley rats- 21 days old | No pretreatment | 132 μg/kg s.c. or 1.2 LD_50_ | Atropine sulphate 2 mg/kg i.m. | HI-6 125 mg/kg i.p. | 660 ± 65 min  of SE during the 24-h period after soman exposure was reported | 57%  survived | Not reported | Apland *et al*., 2018 |

2-PAM= pralidoxime; DZP=diazepam; EEG= electroencephalogram; GD= soman; i.m.= intramuscular; i.p.= intraperitoneal; LD= lethal dose; MDZ=midazolam; s.c= subcutaneous; SE= *status epilepticus;* SRS= spontaneous recurrent seizures
